# Regeneration of Tumor-Antigen-Specific Cytotoxic T Lymphocytes from iPSCs Transduced with Exogenous TCR Genes

**DOI:** 10.1016/j.omtm.2020.09.011

**Published:** 2020-09-20

**Authors:** Takuya Maeda, Seiji Nagano, Soki Kashima, Koji Terada, Yasutoshi Agata, Hiroshi Ichise, Manami Ohtaka, Mahito Nakanishi, Fumihiro Fujiki, Haruo Sugiyama, Toshio Kitawaki, Norimitsu Kadowaki, Akifumi Takaori-Kondo, Kyoko Masuda, Hiroshi Kawamoto

**Affiliations:** 1 Laboratory of Immunology, Institute for Frontier Life and Medical Sciences, Kyoto University, Kyoto 606-8507, Japan; 2 Department of Hematology and Oncology, Graduate School of Medicine, Kyoto University, Kyoto 606-8507, Japan; 3 Department of Urology, Akita University Graduate School of Medicine, Akita 010-8543, Japan; 4 Department of Biochemistry and Molecular Biology, Shiga University of Medical Science, Otsu, Shiga 520-2192, Japan; 5 Biotechnology Research Institute for Drug Discovery, National Institute of Advanced Industrial Science and Technology (AIST), Tsukuba, Ibaraki 305-8568, Japan; 6 Department of Cancer Immunology, Osaka University Graduate School of Medicine, Osaka 565-0871, Japan; 7 Division of Hematology, Rheumatology and Respiratory Medicine, Department of Internal Medicine, Faculty of Medicine, Kagawa University, Kagawa 761-0793, Japan

**Keywords:** adoptive T cell therapy, iPSCs, CD8αβT cells, WT1, TCR

## Abstract

In the current adoptive T cell therapy, T cells from a patient are given back to that patient after *ex vivo* activation, expansion, or genetic manipulation. However, such strategy depends on the quality of the patient’s T cells, sometimes leading to treatment failure. It would therefore be ideal to use allogeneic T cells as “off-the-shelf” T cells. To this aim, we have been developing a strategy where potent tumor-antigen-specific cytotoxic T lymphocytes (CTLs) are regenerated from T-cell-derived induced pluripotent stem cells (T-iPSCs). However, certain issues still remain that make it difficult to establish highly potent T-iPSCs: poor reprogramming efficiency of T cells into iPSCs and high variability in the differentiation capability of each T-iPSC clone. To expand the versatility of this approach, we thought of a method to produce iPSCs equivalent to T-iPSCs, namely, iPSCs transduced with exogenous T cell receptor (TCR) genes (TCR-iPSCs). To test this idea, we first cloned TCR genes from WT1-specific CTLs regenerated from T-iPSCs and then established WT1-TCR-iPSCs. We show that the regenerated CTLs from TCR-iPSCs exerted cytotoxic activity comparable to those from T-iPSCs against WT1 peptide-loaded cell line in *in vitro* model. These results collectively demonstrate the feasibility of the TCR-iPSC strategy.

## Graphical Abstract

**Figure:**
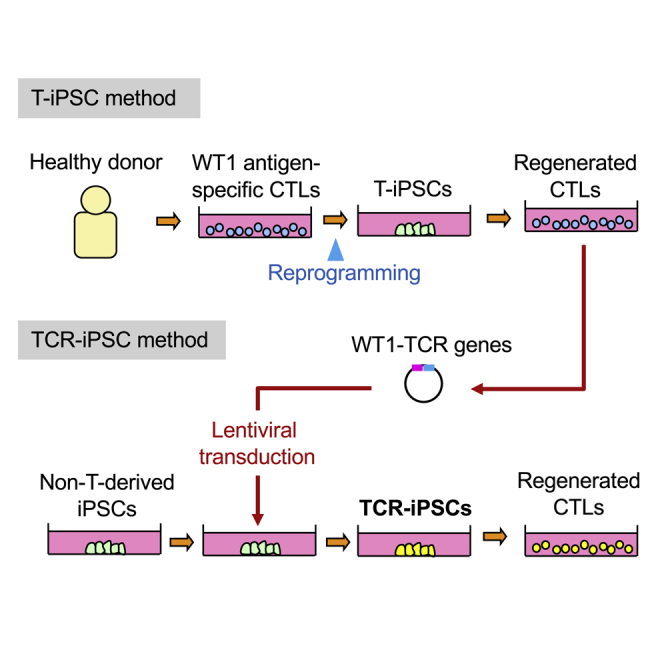

## Teaser

We previously developed a method by which CD8 T cells are regenerated from iPSCs originally derived from CD8 T cells (T-iPSCs). In this study, we transduced iPSCs with exogenous TCR genes and found that the CD8 T cells regenerated from such iPSCs were comparable to the T-iPSC-derived ones in cytotoxicity.

## Introduction

Some adoptive T cell therapies have recently demonstrated remarkable efficacy; for example, T cells forced to express T cell receptor (TCR) genes^1^ or chimeric antigen receptor (CAR) genes^2^^,^^3^ have been shown to be effective therapeutics in certain types of cancer. However, some issues remain to be solved to optimize these strategies. Currently, adoptive T cell therapies are essentially conducted in an autologous setting, where the peripheral T cells are collected from each patient, expanded, and transduced with, for instance, a TCR-expressing retrovirus vector, resulting in a very high cost for each treatment. Another issue is that the quality of the final product depends on the initial quality of the patients’ T cells, which are not good enough in some cases.^4^

To resolve these issues, it would be preferable to conduct adoptive immune cell therapy in an allogeneic setting, where it will be possible to use “off-the-shelf” T cells,^5^, ^6^, ^7^ thus considerably reducing cost and standardizing quality. To make such T cells, several points need to be addressed: (1) such cells should have unlimited expansion capacity; (2) they should not be rejected by the patient’s immune system; and, most importantly, (3) they should be “monoclonal,” because polyclonal T cells inevitably contain dangerous allo-reactive T cells at some frequency.

To achieve such off-the-shelf T cells, we have been developing a method by which T cells can be cloned and expanded using induced pluripotent stem cell (iPSC) technology. Our initial concept was as follows. First, iPSCs are generated from T cells. In such iPSCs, termed T-iPSCs hereafter, the genomic structure of rearranged TCR genes should be inherited by the T-iPSCs and T cells regenerated from these T-iPSCs should express the same TCR as the original T cells.^8^ Considering that there is almost no limit to iPSCs expansion capacity, it should be possible to produce as many “fresh” T cells of a given specificity as needed.

Based on this concept, we succeeded in producing iPSCs from human cytotoxic T lymphocytes (CTLs) specific for the melanoma antigen MART1 and then in regenerating CTLs from MART1-T-iPSCs.^8^ However, later on, we came to notice that the regenerated CTLs in our first paper did not express the CD8αβ heterodimer-like conventional CTLs but instead expressed the CD8αα homodimer, which is less efficient at strengthening the TCR signal. We then made improvements in our culture procedures and succeeded in inducing CD8αβ-type CTLs.^9^ By using this improved method, we regenerated WT1 antigen-specific CTLs. Regenerated CTLs were able to prolong the survival of mice in a xenograft leukemia model, where WT1-expressing human leukemia cells were inoculated into immunodeficient mice followed by transfusion of regenerated WT1-CTLs. In this study, we also showed that such tumor-antigen-specific T-iPSCs can be established from peripheral blood of a healthy donor.

The T-iPSC method described above can be applied in an allogeneic setting. For example, if we produce T-iPSCs from histocompatibility leukocyte antigen (HLA) haplotype-homozygous (HLA-homo) donors, T cells regenerated from such T-iPSCs can be administered to patients who have the same HLA haplotype on at least one of their alleles. However, we still have faced two issues: (1) iPSC clones are very heterogeneous in terms of T-cell-generating potential and (2) the TCR affinity among T-iPSC clones varies greatly. Due to these issues, it is necessary to first produce multiple clones and then stringently select the best one among them. To address these issues, very recently, we have assessed the frequency of “usable” T-iPSC clones among initially established T-iPSC clones.^10^ We found that about 50% of T-iPSC clones were usable in terms of T cell generating potential and cytotoxic activities of regenerated CTLs. We also showed that the regenerated CTLs exhibited allo-reactivity with minimum frequency, indicating that one spare clone is enough when applied in allogenic setting. Taking these points into considerations, we estimated that, in order to reliably obtain two potent T-iPSC clones, it is enough to establish eight initial clones, which we thought is a reasonable number. Nevertheless, it could be pointed out that such method is still time consuming and costly. To solve these issues, we thought of an alternative approach: to transduce iPSCs with an exogenous TCR gene (TCR-iPSC method). Using this method, it would be much easier to establish high-quality TCR-iPSC clones, because we will be able to use iPSCs and TCR genes of guaranteed quality and specificity.

To examine whether this idea would work in practice, it is necessary to compare the quality of T cells expressing the same TCR between those produced by T-iPSC and TCR-iPSC methods. To this aim, in the present study, we cloned WT1-specific TCR genes from CTLs regenerated from WT1-T-iPSCs and transduced non-T-derived iPSCs with these TCR genes (WT1-TCR-iPSCs). Using these WT1-TCR-iPSCs, we succeeded in regenerating CD8αβ CTLs that exhibited antigen-specific cytotoxic activity comparable to CD8αβ CTLs regenerated from T-iPSCs.

## Results

### Transduction of HLA-Homo iPSCs with WT1-TCR Genes and Regeneration of CTLs from the TCR-iPSCs

**Figure 1:**
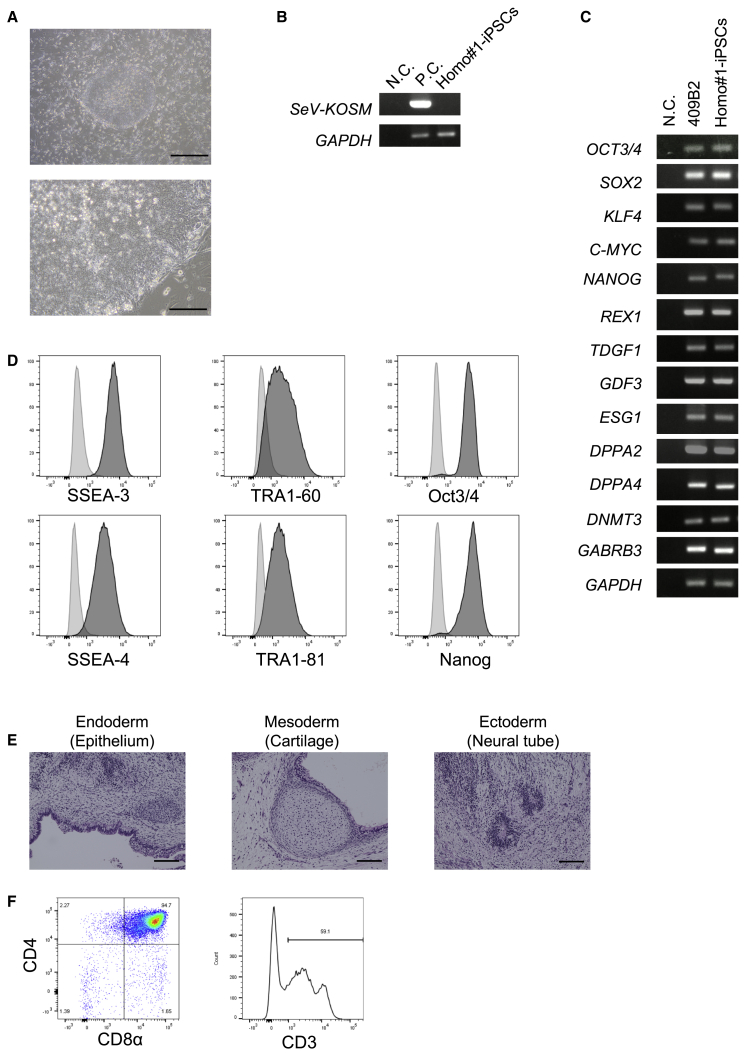
Characterization of Parental HLA-Homo iPSCs (A) A photomicrograph of parental homo #1-iPSCs. Top: ×40, scale bar represents 500 μm; bottom: ×200, scale bar represents 100 μm. (B) Gene expression of the SeV-derived Yamanaka factors (*KOSM* [*KLF4*, *OCT3/4*, *SOX2*, *c-MYC*]) in homo #1-iPSCs. SeV-transfected cells (7 days after infection) were used as a positive control (P.C.). H_2_O was used as a negative control (N.C.). *GAPDH* is an internal control for PCR. (C) The expression of pluripotent stem cell marker genes in homo #1-iPSCs. *GAPDH* is an internal control for PCR. 409B2, human iPSCs established from fibroblasts, were used as a P.C. H_2_O was used as a N.C. (D) Flow cytometric profiles of homo #1-iPSCs stained for indicated antigen (dark gray) and isotype control (light gray). (E) Representative hematoxylin and eosin staining of histological sections of a teratoma derived from homo #1-iPSCs containing tissues derived from all three germ layers. Scale bars represent 100 μm. (F) Flow cytometric profiles of cells regenerated from homo #1-iPSCs on day 44, showing that the parental homo #1-iPSCs retain T-cell-generating potential. Representative of more than three independent experiments is shown.

We first established iPSCs from monocytes derived from the HLA-homo donor (homo #1-iPSCs) as parental iPSCs (Figure 1A) using the Sendai virus system shown in the previous report.^11^ Sendai-virus-derived Yamanaka four factors were not detected in homo #1-iPSCs (Figure 1B). RT-PCR analysis and flow-cytometric analysis showed that these cells express pluripotent stem cell marker genes (Figures 1C and 1D). These iPSCs formed three germ layers in teratoma (Figure 1E). In the previous study, we succeeded in regenerating CTLs from iPSCs.^9^ Using the same method, we confirmed that homo #1-iPSCs efficiently gave rise to CD4/8 double-positive (DP) cells in the T cell induction culture (Figure 1F).

**Figure 2:**
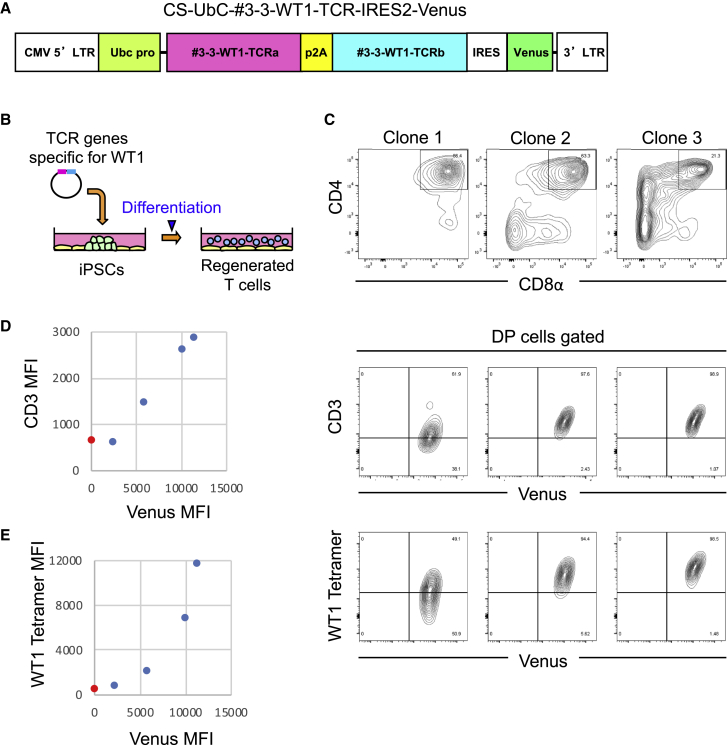
Diversification of TCR Expression Level among TCR-iPSC Clones (A) A map of the lentivirus vector encoding WT1-specific TCR genes and a fluorescent marker Venus gene under the Ubc promotor. cDNAs encoding the TCR α and β chain were combined with a P2A sequence to ensure equal amounts of TCR α and β chain protein expression. (B) A schematic illustrating the TCR-iPSC method. iPSCs were transduced with the lentiviral vector described in (A) containing TCR genes specific for WT1 and then differentiated into CD8 T cells. (C) Flow cytometric profiles of DP cells differentiated from Venus low (left), middle (middle), and high (right) clones. Representative of three independent experiments is shown. (D and E) Graphs indicate Venus versus CD3 (D) or WT1 tetramer (E) mean fluorescent intensity (MFI) of CD4/8 DP cells derived from various TCR-iPSC clones measured by flow cytometry as (C). Red dots indicate the MFI of the non-transduced control. Representative of three independent experiments is shown.

Next, we cloned WT1-specific TCR α and β chain genes from WT1-specific CTLs regenerated from #3-3-WT1-T-iPSCs,^9^ which will be hereafter referred as “#3-3-WT1-TCR genes.” #3-3-WT1-TCR genes were incorporated into a lentivirus vector together with the Venus gene as a marker. We chose lentivirus and the Ubc promotor system to transfer TCR genes because of the less-frequent silencing of transduced genes during long-term culture of transduced iPSCs (Figure 2A). Then, we transduced the homo #1-iPSCs with #3-3-WT1-TCR genes, and Venus-positive colonies were picked up as transduced clones, from which T cells were regenerated (Figure 2B). Among T cell clones regenerated from several TCR-iPSC clones, we show examples of the expression profile of CD3 versus Venus and that of WT1-tetramer versus Venus in DP cells generated from three different clones, which represent Venus-low-, medium-, and high-expressing cells, respectively (Figure 2C). TCR expression levels by DP cells derived from these clones correlated well with Venus intensity levels (Figure 2D). The WT1 tetramer levels, which are considered to reflect TCR avidity, also correlated well with Venus levels (Figure 2E). Therefore, we decided to use one of the Venus high clones, namely #3-3-WT1-TCR-Homo #1-1-iPSCs (hereafter referred to as #3-3-WT1-TCR-iPSCs). The numbers of integration copies of the WT1-TCR in each clone estimated by the method shown in Materials and Methods were 1, 2, and 4 for Venus-low, medium, and high clone, respectively (Table 1).

**Table 1:** Estimation of the Copy Numbers of Transduced TCR Genes

	Venus Copy Numbers/Cell	WPRE Copy Numbers/Cell
Venus low	1.56	1.32
Venus medium	2.28	2.08
Venus high	3.66	3.70

### The Cytotoxic Activity of Regenerated CD8 T Cells from TCR-iPSCs Was Comparable to Those from T-iPSCs

**Figure 3:**
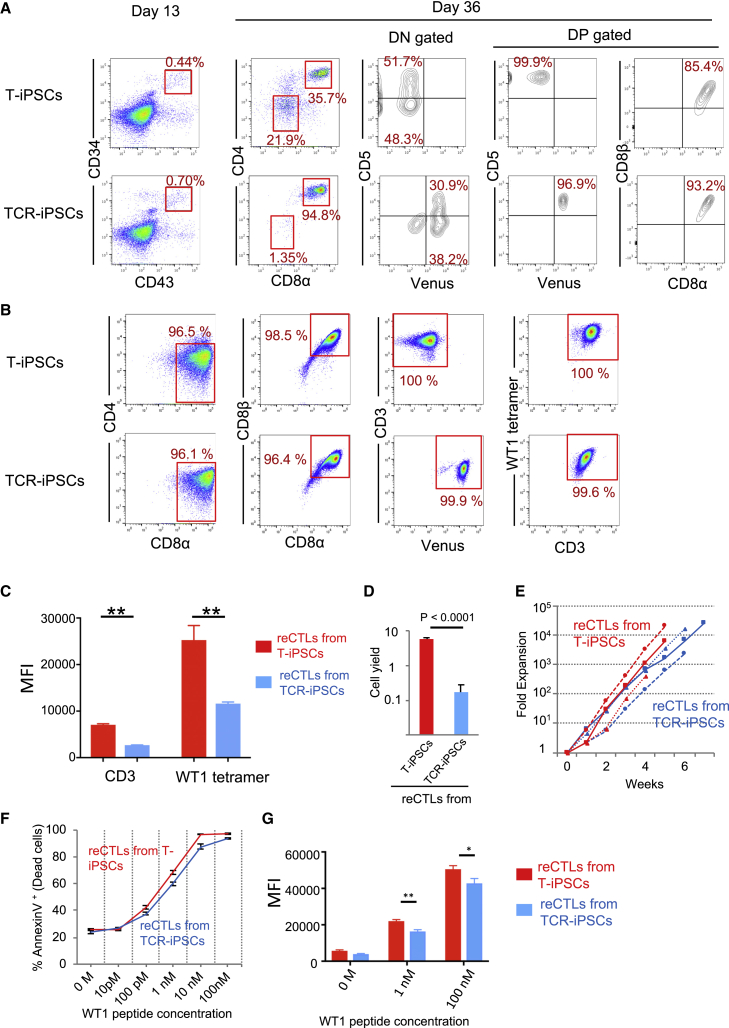
Growth Potential and Cytotoxic Activity of WT1-Specific Regenerated CTLs from TCR-iPSCs (A) Flow cytometric profiles of hemangioblasts on day 13 and immature T cells on day 36 generated from T-iPSCs (#3-3-WT1-T-iPSCs) and TCR-iPSCs (#3-3-WT1-TCR-iPSCs). Representative of more than three independent experiments is shown. (B) Flow cytometric profiles of mature CD4^−^CD8^+^ (CD8) T cells generated from T-iPSCs (#3-3-WT1-T-iPSCs) and TCR-iPSCs (#3-3-WT1-TCR-iPSCs). Representative of more than three independent experiments is shown. (C) A graph indicates the MFI of CD3 and WT1 tetramer of regenerated CD8 T cells from T-iPSCs (#3-3-WT1-T-iPSCs) and TCR-iPSCs (#3-3-WT1-TCR-iPSCs). n = 3; ∗∗p < 0.01. (D) Cell yield of CD8 T cells compared with DP cells at the induction step from DP to CD8 T cells. DP cells were cultured with LCL pulsed with WT1 peptide for 6 days and analyzed by flow cytometry. Cell yield was calculated by dividing the number of CD8αβ SP cells with the number of starting DP cells. Data are shown as mean ± SD. Statistical comparisons were performed using an unpaired two-tailed Student’s t test. Representative of more than three independent experiments is shown. (E) Fold expansion of regenerated CTLs (reCTLs) by repeated stimulation. reCTLs were stimulated every week by co-culturing with LCLs pulsed with WT1 peptide (100 nmol/L). Plot shows the data of three independent experiments. Red, reCTLs from T-iPSCs (#3-3-WT1-T-iPSCs); blue, reCTLs from TCR-iPSCs (#3-3-WT1-TCR-iPSCs). (F) Comparison of the cytotoxic activity of reCTLs derived from T-iPSC and TCR-iPSC against C1R-A∗24:02 cells loaded with WT1 peptide. All experiments were performed using the regenerated CD8 T cells cultured for 3–5 weeks. The effector-to-target (E:T) ratio was fixed at 3:1. Data are presented as mean ± SD. Representative of three independent experiments is shown. (G) A graph indicates MFI of IFNγ of regenerated CD8 T cells from T-iPSCs (#3-3-WT1-T-iPSCs) and TCR-iPSCs (#3-3-WT1-TCR-iPSCs) co-cultured with WT1-peptide-pulsed LCL for 4 h. E:T = 1:1. Data are presented as mean ± SD. Representative of two independent experiments is shown. ∗p < 0.05; ∗∗p < 0.01.

We differentiated T cells from #3-3-WT1-TCR-iPSCs and from #3-3-WT-1-T-iPSCs in parallel as previously described (Figure 3A). On day 13, CD43^+^CD34^+^ cells representing hemangioblasts were similarly formed in both cultures. On day 36, CD4^+^CD8^+^ DP cells representing immature T cells were generated in both cultures. Although proportion of DN cells looked different between two groups, this difference is not so essential because, in general, DN versus DP ratio greatly changes during cultivation. Approximately half of DN cells express CD5 in both groups, and DP cells from T-iPSCs and TCR-iPSCs were found to be identical in that they were exclusively CD8αβ^+^ and CD5^+^. These DP cells were then enriched by using CD4 MicroBeads and stimulated them with B lymphoblastoid cell line (LCL) pulsed with WT1 peptide, as previously published, to induce mature CD4^−^CD8^+^ (CD8) T cells.^9^ The regenerated cells from both T-iPSCs and TCR-iPSCs were uniformly CD4^−^CD8^+^ and expressed the CD8αβ heterodimer, Venus, and the WT1-specific TCR (Figure 3B). The expression level of CD3 and the WT1 tetramer binding intensity to CD8 T cells derived from TCR-iPSCs was slightly lower than that from T-iPSCs, probably reflecting lower expression of transgenes compared with endogenous genes (Figures 3B and 3C). The efficiency of CD8 T cell generation in the induction process from DP cells, measured as cell yield of CD8 single positive (SP) compared with starting DP cells, was much lower in TCR-iPSC case (Figure 3D). Whether this low efficiency results from the low level of TCR expression or the intrinsic heterogeneity of the iPSC line in terms of developmental potential remains unclear. However, once generated, the expansion of CD8 T cells derived from TCR-iPSCs and T-iPSCs was comparable (Figure 3E). The antigen-specific cytotoxicity and interferon γ (IFNγ) production of TCR-iPSC-derived CD8 T cells was also almost the same as that of T-iPSC-derived CD8 T cells (Figures 3F and 3G).

### Regenerated CD8 T Cells Were Virtually Monoclonal

**Figure 4:**
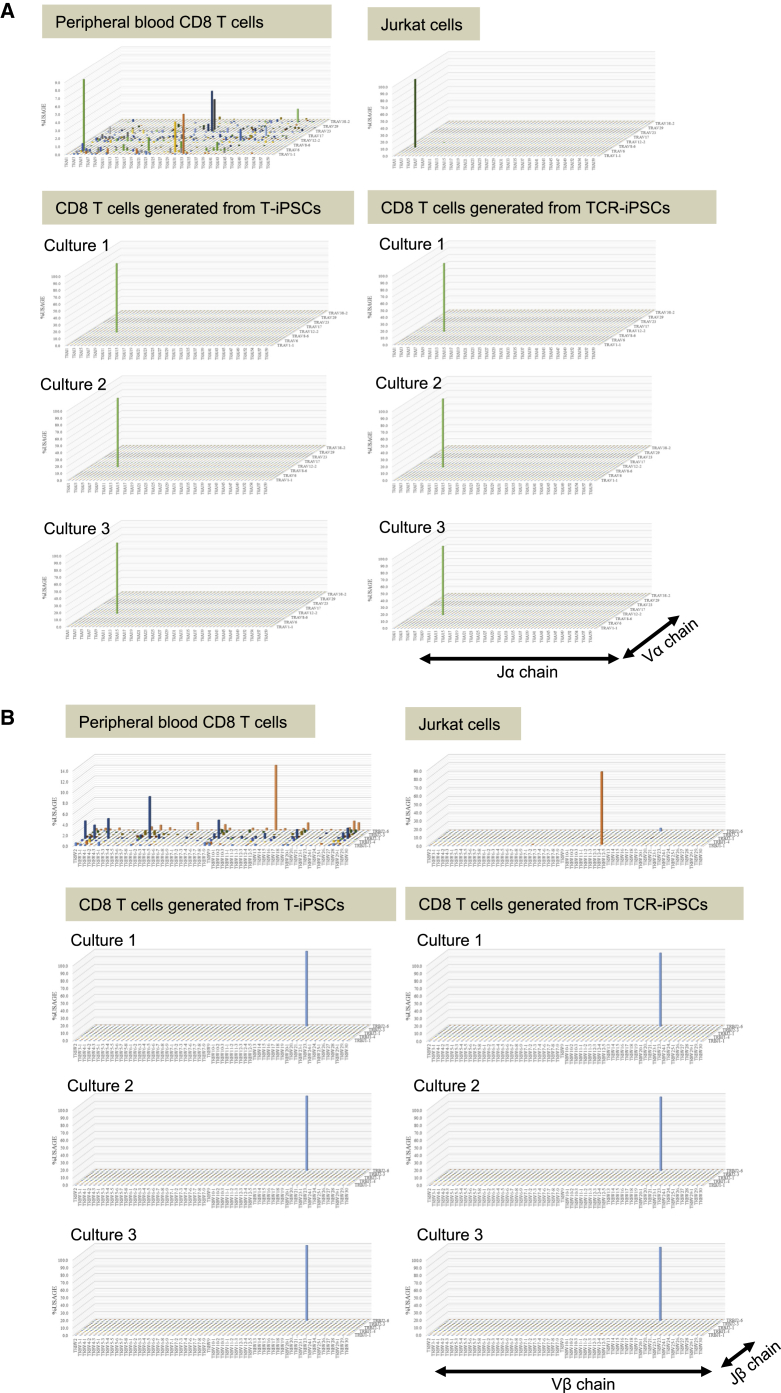
TCR Repertoire Analysis of Regenerated CD8 T Cells Demonstrates Their Monoclonality Peripheral blood T cells collected from a healthy donor, Jurkat cells, regenerated CD8 T cells from T-iPSCs (#3-3-WT1-T-iPSCs) and those from TCR-iPSCs (#3-3-WT1-TCR-iPSCs) were analyzed for specific CDR3 amino acid sequences. (A) Usages of TCRα chain variable (TRAV) and joining (TRAJ) region genes are shown in Z axis by percentage. (B) Usages of TCRβ chain variable (TRBV) and joining (TRBJ) region genes are shown in Z axis by percentage.

In our method, WT1 peptide-pulsed LCLs were used for the induction of CD8 T cells from DP cells and for the subsequent expansion of CD8 T cells. Therefore, CD8 T cells after expansion were almost exclusively WT1-tetramer^+^ cells. We investigated TCR gene usage by next-generation sequencing of regenerated T cells in three independent cultures derived from T-iPSCs and TCR-iPSCs. The repertoires of regenerated T cells derived from T-iPSCs and TCR-iPSCs were found to be virtually completely monoclonal for both α and β chains (Figure 4; Table 2).

**Table 2:** Sequencing Reads of TCR Genes

	TCRα Total Reads	TCRα in Flame	TCRβ Total Reads	TCRβ in Flame
PB CD8	188,725	128,318	180,245	112,607
Jurkat	86,326	62,569	157,625	10,434
T-iPSC culture 1	137,520	83,776	130,988	95,772
T-iPSC culture 2	147,146	96,148	185,628	124,144
T-iPSC culture 3	134,389	89,185	133,916	106,803
TCR-iPSC culture 1	114,949	45,651	230,728	14,005
TCR-iPSC culture 2	137,574	54,363	223,765	14,766
TCR-iPSC culture 3	162,739	60,527	276,781	13,345

## Discussion

In the present study, we have succeeded in producing potent CTLs from iPSCs transduced with exogenous TCR genes. We expect that this technology will be a key method for the preparation of off-the-shelf T cells.

This novel method has some significant advantages over the conventional T-iPSC method. First, it becomes possible to use a high-quality, cancer-antigen-specific TCR that has already been well characterized in terms of efficacy and safety. The quality of a TCR is determined by its affinity and specificity for the target antigen. In general, higher affinity is preferable, but the affinity of TCRs in T cells collected from patients is usually not so high, because high-affinity TCRs have been mostly eliminated by various self-tolerance mechanisms. Thus, many efforts have been made in the cancer immunotherapy field to raise TCR affinity by genetic engineering.^12^ On the other hand, for the sake of safety, the TCR specificity is critical. Therefore, a TCR with high affinity for the cancer antigen while having no cross-reactivity to other antigens is ideal.

As to the safety issue, in the case of the T-iPSC setting, the TCR being used was derived originally from the patient, and so it should basically be safe when applied in an autologous setting. The allogeneic TCR may exert the so-called allo-reaction against the peptide presented on the mismatched major histocompatibility complex (MHC), leading to “off target, off tumor” side effects.^13^^,^^14^ One should be even more careful about off target, off tumor side effects when a genetically engineered TCR is used,^15^^,^^16^ because such an artificially altered TCR has not undergone negative selection in the body, a physiological process to eliminate autoreactive T cells. Taking these issues into consideration in designing a TCR-iPSC strategy, it is desirable to use a TCR where the efficacy and safety for use in an allogeneic setting have been clinically demonstrated.

The second advantageous point of our TCR-iPSC strategy is that high-quality iPSCs can be used as parental cells. The quality of iPSCs can be evaluated based on how close they are to embryonic stem cells (ESCs) in terms of the number of mutations and the epigenetic status of the genome^17^^,^^18^ and, in our case, additionally their efficiency to differentiate into T cells.^10^^,^^19^ In that sense, in Japan, high-quality iPSCs are being stored as “iPSC stocks.” These iPSCs are produced from healthy donors who are homozygous for an HLA haplotype; the regenerated cells are expected to be transferred into patients who share that HLA haplotype on one allele. At present, the four most-frequent HLA haplotype lines are available, covering around 35% of Japanese people.^20^ On the other hand, as an alternative approach, it is also possible to use ESCs as parental cells. To avoid immunological rejection in the case of ESCs, MHC class I and II genes could be deleted.^21^^,^^22^ By the use of such MHC-null ESCs, it should become possible to transfuse regenerated cells into any patient, although possible immune reactions by NK cells due to “missing self-recognition”^11^^,^^23^ or against minor histocompatibility antigens remain a concern.

In this study, we used a lentivirus system to transduce iPSCs with TCR genes and the Venus fluorescent protein as marker and showed that Venus intensity can be used as surrogate marker for the transduction efficiency and expression level of TCR genes. However, even when we selected clones expressing the highest Venus level, the differentiated T cells expressed lower TCR levels compared to parental T-iPSC-derived T cells. There are several reports that the transduction of TCRs or even CAR genes into the TCR gene locus results in better control of expression than does random integration by the virus vector because of the physiological regulation of these exogenous genes in the TCR loci.^24^^,^^25^ Such TCR-locus-specific transduction in iPSCs would solve the problems of levels and regulation of TCR transcripts inherent in the TCR-iPSC method.

Recently, another group described T cell differentiation from TCR-transduced iPSCs using similar strategy as ours.^26^ In that report, it was shown that CTLs regenerated from TCR-transduced iPSCs were potent effector cells in *in vitro* and *in vivo* xenograft models. However, because there was no direct comparison in that study between CTLs expressing the same TCR produced by T-iPSC or TCR-iPSC technology, the actual feasibility of the TCR-iPSC method remains unstudied. In the present study, we directly compared the T-iPSC and TCR-iPSC methods and found that the TCR-iPSC method was almost equivalent in terms of cytotoxic activity and in proliferation in an *in vitro* setting, although there was some difference in T cell differentiation efficiency. They also showed that deletion of the *RAG2* gene served to prevent loss of target specificity. However, we showed that, by specific activation using antigen-presenting cell (APC) pulsed with cognate peptide, regenerated T cells are virtually clonal both with the T-iPSC and TCR-iPSC methods by next-generation TCR sequencing, which is sensitive enough to detect TCRs of 0.1%–0.01% frequency according to the published patent data (US20160289760A1). For ensuring more safety, *RAG* gene deletion is ideal, but it is not mandatory.

Up to this point, we have discussed the strategy to prepare off-the-shelf T cells by using the TCR-iPSC method. However, we envision that the TCR-iPSC method can also be applied to personalized medicine approaches. Recently, so-called “neoantigens” have been attracting attention in the field of cancer immunotherapy.^27^^,^^28^ Tumor cells that have accumulated somatic mutations that change amino acids in the encoded proteins may express proteins that can be recognized by each patient’s immune system as foreign neoantigens. Such neoantigens could be ideal cancer antigens, because they are expressed exclusively by tumor cells. In line with this idea, methods to use TCR genes specific for neoantigens in TCR gene transfer therapy^29^ are being developed.

In order to target neoantigens by our approach, we plan to transduce allogeneic iPSCs or ESCs with neoantigen-specific TCR genes collected from each cancer patient. By doing so, multiple clones of regenerated CTLs could be produced, and a CTL cocktail prepared by mixing these CTLs clones could be transfused into the patient. In such a setting, it can be said that, although the T cells are allogeneic, the TCRs are autologous.

## Materials and Methods

### Study Approval

This study was approved by the institutional review board of the Graduate School of Medicine, Kyoto University (approval number: G761) and abided by the tenets of the Declaration of Helsinki. All specimens from healthy individuals and patients were collected after written informed consent was obtained.

### Cell Lines

OP9 and OP9/DLL1 were purchased from RIKEN BRC. C1R-A∗24:02 was a gift from Dr. Masafumi Takiguchi (Kumamoto University). An autologous LCL was established from peripheral blood of a healthy donor from whom #3-3-WT1-T-iPSCs were established as described previously.^9^ After obtaining the cell lines, frozen stocks were prepared within one to five passages and new stocks were thawed frequently to maintain the original condition. The cell lines were passaged for less than 3 months after receipt or resuscitation. They were also authenticated by morphology, growth rate, and surface phenotype, especially by the expression of HLA class I.

### Establishment of Monocyte-Derived iPSCs from HLA-Homo Donor

iPSCs derived from monocytes were established by the previously reported method with slight modifications.^11^^,^^30^ Briefly, peripheral blood mononuclear cells (PBMCs) from the HLA-homo donor were isolated using Ficoll-Paque PLUS (GE Healthcare) and CD14^+^ monocytes were enriched by positive selection using CD14 MicroBeads (Miltenyi Biotec). 1 × 10^6^ cells were transduced with Sendai virus vector containing the four Yamanaka factors. Following 2 h incubation at 37°C, cells were seeded onto murine embryonic fibroblast (MEF) feeder cells and cultured in RPMI-1640 supplemented with 10% human AB serum. From at day 2, half of the medium was replaced with human iPSC medium, Repro Stem (ReproCELL) supplemented with 5 ng/mL basic fibroblast growth factor (bFGF) (Wako Pure Chemicals Industries). Each iPSC colony that appeared between day 20 and 35 was picked up and expanded as iPSC clone.

### Gene Expression Analysis by RT-PCR

Total RNA was isolated using RNeasy Plus Mini Kit (QIAGEN), and cDNA was synthesized according to the manufacturer’s protocol using a SuperScript VILO cDNA Synthesis Kit (Thermo Fisher). cDNA was amplified by PCR using various sets of primers as described.^8^ 409B2, a human iPSC established from fibroblasts, was used as a positive control.

### Teratoma Formation Assay

All mouse experiments were performed following the accordance with the Institutional Animal Care and Use Committee regulations of Kyoto University. 1.0 × 10^6^ human iPSCs were injected into the testis of non-obese diabetic (NOD)/ShiJic-severe combined immunodeficiency (SCID) mice. 12 weeks after injection, tumors were resected, fixed in 1% formaldehyde, and embedded in paraffin. Sections were stained with hematoxylin and eosin.

### Flow Cytometry

The following monoclonal antibodies were used: CD34 (8G12); CD43 (1G10); CD3 (UCHT1); CD4 (RPAT4); CD5 (UCTH-2); CD8α (HIT8a); CD8β (2ST8.5H7); SSEA-3 (MC-631); SSEA-4 (MC813-70); TRA1-60 (TRA-1-60); TRA1-81 (TRA-1-80); Oct3/4 (40/Oct-3); Nanog (N31-355); and IFNγ (4S.B3). All antibodies (Abs) were purchased from BioLegend or BD Biosciences. HLA-A∗24:02^+^ WT1 (235–243 amino acids [aas] CYTWNQMNL) tetramers (MBL International) were used for the detection of T cells expressing TCR specific for WT1. Flow cytometry was performed using a FACS CantoII with FlowJo software (Tree Star).

### Cloning of WT1-Specific TCR α and β Chain Genes and Construction of the WT1 TCR Lentivirus Vector

WT1-specific TCR α and β chain genes of regenerated CTL derived from #3-3-WT1-T-iPSC were cloned using a 5′ rapid amplification of cDNA ends (RACE) method. Each cDNA encoding TCR α and β chain genes was linked with the self-cleaving sequence P2A and subcloned into pENTR/D-TOPO vector and further subcloned into an expression vector, CS-UbC-RfA-IRES2-Venus (kindly provided from Dr. Atsushi Miyawaki [RIKEN] and Dr. Hiroyuki Miyoshi [Keio University]) using pENTR Directional TOPO Cloning Kits (Thermo Fisher Scientific).

### Transduction of the WT1 TCR Lentivirus Vector to HLA-Homo iPSCs

Lentiviruses were collected 48–72 h after transfection of Lenti-X 293T cells (Clontech Laboratories) with appropriate amounts of lentiviral vectors, pRSV-Rev, pMDLg/pRRE, and pMD2.G (Addgene) using ViaFect (Promega). 5 × 10^4^ iPSCs were infected by centrifugation and seeded onto MEF with iPSC medium. Venus-expressing iPSC colonies were picked up manually and maintained in iPSC medium.

### Differentiation of T-iPSCs or TCR-iPSCs into CD8αβ Single-Positive Cells

T-iPSCs were differentiated into CD4/8 DP cells using the OP9 and OP9/DLL1 stromal cell co-culture systems as described,^9^ with slight modification. In brief, iPSC colonies were dissociated using trypsin (0.25%) and collagenase IV (1 mg/mL) and mechanically disrupted into small clumps by pipetting. About 600 iPSC clumps were collected and plated on gelatin pre-coated OP9 dishes filled with OP9 medium, i.e., α-MEM (minimum essential medium) (Invitrogen) with 20% fetal calf serum (FCS). On day 13, colonies were treated with collagenase type IV (50 U/mL) and trypsin-EDTA (0.05%). Cells were plated on an OP9/DLL1 semi-confluent dish in OP9 medium containing hIL-7 (5 ng/mL), hFlt-3L (5 ng/mL), and hSCF (5 ng/mL). On day 15, semi-adherent cells were collected and passage into a new dish layered with OP9/DLL1 cells. From this point, passage was done every 7 days. On day 40, floating cells were collected and CD4/8 DP cells were enriched by using CD4 MicroBeads (Miltenyi Biotec). DP cells were stimulated with HLA-A∗24:02^+^ LCL pulsed with the WT1 peptide (235–243 aas CYTWNQMNL) in the presence of hIL-7 (5 ng/mL) and hIL-21 (10 ng/mL) to induce CD8 T cells. To expand CD8 T cells, cells were stimulated one to five times by HLA-A∗24:02^+^ LCL pulsed with the WT1 peptide in the presence of hIL-7 (5 ng/mL) and hIL-21 (10 ng/mL).

### Annexin V Cytotoxicity Assays

C1R-A∗24:02 used as target cells were labeled with 5(6)-CFDA N-succinimidyl ester (CFSE) (1 μg/mL) and pulsed with WT1 peptide at various concentrations. Target cells and effector cells were co-cultured at an effector-to-target (E:T) ratio of 3:1 in 96-well V-bottomed plates (Nunc) for 6 to 12 h. Dead and dying cells were detected with the combination of annexin V (BioLegend) and phosphatidylinositol (PI). Annexin-V-positive cells in the CFSE-positive population were defined as dead and dying cells. All experiments were performed using the regenerated CD8 T cells cultured for 3–5 weeks with stimulation.

### Intracellular Cytokine Assay

LCL pulsed with WT1 peptide at 0, 1, and 100 nM were used as target cells. 1 × 10^5^ cells of target and effector cells were co-cultured for 4 h in the presence of monensin (GolgiStop; BD Biosciences) in 96-well round bottom plate. Cells were stained with antibody against CD8a, followed by performing the intracellular staining with anti-IFNγ antibody using Foxp3/Transcription Factor Staining Buffer Kit (Tonbo Biosciences).

### Estimation of the Copy Numbers of Transduced TCR Genes

Genomic DNA was prepared by the standard proteinase K method. To estimate the copy numbers of the WT1-TCR gene, we measured the copy numbers of the Venus gene and woodchuck hepatitis virus post-transcriptional regulatory element (WPRE) region, which are included in the CS-UbC-#3-3-WT1-TCR-IRES2-Venus lentiviral vector (12,457 bp) and integrated into the genome upon viral transduction along with the TCR gene. Amounts of the Venus gene, WPRE region, and human CD14 gene as an internal control^30^ were quantified by real-time PCR using QuantiTect SYBR Green PCR mix (QIAGEN) on CFX384 Real-Time System (Bio-Rad). To generate standard curves for the Venus gene and WPRE region, CS-UbC-#3-3-WT1-TCR-IRES2-Venus plasmid DNA equivalent to 40,000 copies (0.56 pg) was mixed with genomic DNA equivalent to 10,000 copies (30 ng corresponding to 5,000 cells based on the assumption that 3 × 109 bp of haploid genome DNA corresponds to 3 pg) prepared from non-transduced iPSCs and serially diluted to 40,000, 20,000, 10,000, and 5,000 copies per PCR reaction. Amounts of the Venus gene and WPRE region of genomic DNA equivalent to 5,000 cells (30 ng) prepared from WT1-TCR transduced iPSCs were quantified by real-time PCR and calculated from standard curves, followed by normalization with amounts of CD14 gene. PCR amplification of Venus and WPRE was performed with the following primers: *Venus*, 5′-TGCCCGACAACCACTACCTG-3′ and 5′-CGATAAGCTTGATCCCTCGATG-3′; *WPRE*, 5′-CCTCAATCCAGCGGACCTTC-3′ and 5′-TTGCTACTTGTGATTGCTCCA-3′, and PCR amplification of *CD14* was performed with primers as described.^31^

### NGS of TCR Repertoire

CD8 SP cells were sorted from PBMCs of a healthy donor as CD3^+^ CD8^+^ CD4^−^ cells by BD AriaIII. Regenerated CTLs from T-iPSC or TCR-iPSC were expanded three times by LCL pulsed with peptide. Total RNA was extracted from each sample by RNeasy Mini Kit (QIAGEN) according to the manufacturer’s instructions. Sequencing of the TCRα/β was performed at Repertoire Genesis (Osaka, Japan) using the unbiased gene amplification method with Adaptor-Ligation PCR.^32^ About 10^5^ valid reads were generated (Table 1). Bioinformatics analysis was then performed using the repertoire analysis software, Repertoire Genesis (RG), provided by Repertoire Genesis (Osaka, Japan). RG assigns TCRα chain variable (TRAV), TCRα chain joining (TRAJ), TCRβ chain variable (TRBV) and TCRβ chain joining (TRBJ) alleles to queries and then generates CDR3 sequences, finally aggregating their combination patterns. Out-of-frame sequences were excluded from the analyses.

## Author Contributions

T.M., K.M., A.T.-K., and H.K. conceived and designed the project. T.M., S.N., S.K., K.T., Y.A., M.O., and K.M. performed the experimental work. F.F., H.S., T.K., and N.K. gave insightful comments in designing the experiments and interpreting data. T.M., K.M., and H.K. wrote the manuscript. All authors discussed the results.

## Conflicts of Interest

The authors declare no competing interests.
